# Health of white sucker within the St. Louis River area of concern associated with habitat usage as assessed using stable isotopes

**DOI:** 10.1007/s10646-013-1167-5

**Published:** 2013-12-27

**Authors:** V. S. Blazer, J. Hoffman, H. L. Walsh, R. P. Braham, C. Hahn, P. Collins, Z. Jorgenson, T. Ledder

**Affiliations:** 1 Leetown Science Center, Fish Health Branch, U.S. Geological Survey, 11649 Leetown Road, Kearneysville, WV 25430 USA; 2 Mid-Continent Ecology Division, U.S. Environmental Protection Agency, 6201 Congdon Blvd, Duluth, MN USA; 3 College of Agriculture and Forestry, West Virginia University, P.O. Box 6125, Morgantown, WV 26506 USA; 4 Minnesota Department of Natural Resources, 1201 E. Hwy 2, Grand Rapids, MN USA; 5 Twin Cities Ecological Services Field Office, U.S. Fish and Wildlife Service, 4101 American Boulevard East, Bloomington, MN 55425 USA; 6 Lake Superior National Estuarine Research Reserve, University of Wisconsin, Superior, WI USA

**Keywords:** White sucker, Stable isotopes, Habitat usage, Tumors

## Abstract

In Spring 2011, 200 adult white sucker were collected in four areas of the St. Louis River area of concern (AOC), located in Minnesota and Wisconsin, USA. The areas included the upper AOC as a reference area, the upper estuary, St. Louis Bay and Superior Bay. Grossly visible abnormalities were documented and preserved for microscopic analyses, as were five to eight representative pieces of liver tissue. A piece of dorsal muscle was preserved for stable isotope analyses and otoliths removed for age determination. The incidence of raised skin lesions (mucoid plaques) was high (31 %), however, microscopically only 4.5 % of the white suckers had neoplasia (papillomas). The remaining lesions were epidermal hyperplasia. Superior Bay had the lowest percentage of skin/lip lesions (10 %), while St. Louis Bay had the highest (44 %). St. Louis Bay also had the highest incidence of skin neoplasms (12 %). No hepatocellular neoplasms were documented, however bile duct tumors were observed in 4.5 % of the suckers. Foci of cellular alteration were observed in fish from all sites except the upper AOC. Stable isotope data indicated that most of the suckers relied on the St. Louis River AOC for the majority (>75 %) of their diet, indicating they were resident within the AOC and not in Lake Superior. The amount of diet obtained from the upper estuary was a significant predictor of skin lesion incidence. Hence, habitat use within the AOC appears to be an important risk factor for skin and possibly, liver lesions.

## Introduction

The utility of effects-based monitoring to assess ecosystem health in the aquatic environment has received increasing attention in recent years. Chemical and other stressor-based monitoring compares the observed concentrations of specific contaminants (or other stressors) with concentrations known to produce effects. However, chemical analyses of water and sediment are a snap shot in time and considerable variation in presence and concentration, spatially within a site and temporally (seasonally), have been demonstrated (Bay et al. 2003; Hamers et al. 2003; Martinović et al. 2008; Lee et al. 2012). Conversely, fish and other aquatic organisms are exposed to complex mixtures of stressors throughout their life span. Stressors include infectious agents, parasites, water quality (e.g. dissolved oxygen, pH, temperature), as well as legacy contaminants and chemicals of emerging concern (pharmaceuticals, current use pesticides, natural and synthetic hormones) that may have additive, synergistic and/or antagonistic effects (Meek et al. 2011; Barber et al. 2013). In addition, both abiotic and biotic (predation, parasite infestations, infectious agents) factors can modulate the effects of anthropogenic contaminants on populations (Schwaiger 2001; Jobling and Tyler 2003; Marcogliese and Pietrock 2010; Fischer et al. 2013). Organisms living in a particular habitat integrate the effects of these stressors over time and may have adaptive or compensatory responses or demonstrate adverse effects. For all of the above reasons, biomarkers of effect, that measure the functional capacity of an organ system or whole organism (Huggett et al. 1992; Shugart et al. 1992; Depledge and Galloway 2005; Connon et al. 2012) can be very cost-effective indicators of adverse effects that will direct more in-depth studies of risk factors and cause(s). While assessing effects in resident species provides highly representative information for a site, numerous factors such as age, species sensitivity, feeding habits and movement are important, particularly when using a fish species. One concern in effects-based monitoring programs is whether the effects noted are reflective of the capture site habitat.

The carbon (C) or nitrogen (N) stable isotope composition, ^13^C:^12^C or ^15^N:^14^N, of fish tissue is a time-integrated marker of the fish’s diet. The average difference between the C and N stable isotope composition (denoted as δ^13^C and δ^15^N, respectively) of a consumer and its recent diet is ±0.4 % δ^13^C and ±3.4 % δ^15^N for whole organisms and muscle tissue (Vander Zanden and Rasmussen 2001). In adult fish, the isotopic turnover time (i.e. the time required for a consumer to resemble the isotopic composition of its prey) is 6 months to 2 years due to a combination of catabolic and metabolic processes (Hesslein et al. 1993; Weidel et al. 2011). Where the isotope composition of prey differs among locations, it can be used to characterize fish movement or site fidelity (Hoffman et al. 2010) and hence habitat usage. Stable isotope analyses have been used as indicators of anthropogenic effluent sources (Hoffman et al. 2012), anthropogenic effects on fish assemblages (Freedman et al. 2012) and rarely in association with fish health monitoring (Schlacher et al. 2007).

Gross external abnormalities and liver diseases, particularly of benthic fishes, have been widely used as effects associated with the presence of environmental contaminants (Myers et al. 1994; Vethaak and Jol 1996; Vethaak and Wester 1996; Fournie et al. 1996; Feist et al. 2004). Liver and skin tumors in brown bullhead (*Ameiurus nebulosus*) and white sucker (*Catostomus commersonii*) in the Great Lakes and other geographical areas are associated with environmental degradation (Baumann 1992; Baumann et al. 1996; Rafferty et al. 2009; Pinkney et al. 2011). For this reason, “fish tumors or other deformities”, was one of the beneficial use impairments (BUIs) identified by the International Joint Commission (IJC) at Areas of Concern (AOC) by the U.S.–Canada Great Lakes Water Quality Agreement, Annex 2 of the 1987 Protocol (IJC 1989). Areas of Concern are defined as “geographic areas that fail to meet the general or specific objectives of the agreement where such failure has caused or is likely to cause impairment of beneficial use of the area’s ability to support aquatic life.” Within the AOC framework, 14 Beneficial Use Impairments (BUIs) are defined; the presence of one or more suggesting the location has experienced environmental degradation (http://www.epa.gov/glnpo/aoc). The 2010 Great Lakes Restoration Initiative, a multi-agency, multi-year effort targeted specific priorities including evaluation and monitoring progress in AOCs within the Great Lakes region. Although the fish tumor BUI remains at numerous AOC, in many cases such as the St. Louis River AOC, the listing was based on unsubstantiated observations and there is not sufficient data to evaluate the current status (http://www.epa.gov/glnpo/aoc/stlouis/StLouis-AOC_BUIs.pdf). There is interest in determining if the St. Louis AOC can be delisted in regards to this particular BUI or if further remediation is necessary.

White sucker are abundant, widespread in North America and have been used as an indicator species for contaminant effects-based monitoring at numerous geographical locations (Munkittrick and Dixon 1989; Servos et al. 1992; Woodling et al. 2006; Bowron et al. 2009; Miller et al. 2009). An increased prevalence of skin and liver tumors of white sucker has been documented at contaminated sites within the Great Lakes drainage, when compared to less contaminated sites (Sonstegard 1977; Smith et al. 1989; Hayes et al. 1990; Baumann 1992; Premdas et al. 1995; Baumann et al. 1996). However, definitive data on risk factors for liver and skin neoplasms in white sucker is lacking. Catostomids, including white sucker are known to migrate, sometimes long distances, to appropriate stream spawning habitat in the spring. They may move between rivers and lakes, and hence, their utility as an indicator species for a specific area has been questioned. For this reason it is important to know the life history of a specific population and habitat usage during the majority of its lifespan. Doherty et al. (2010) used stable isotopes and radio/acoustic tracking to determine movements of white sucker in the Saint John River in Canada. They found white sucker maintain a high fidelity to well-defined reaches of the river outside of the spawning period, reflect localized environmental conditions and can be used as sentinel species.

The lower St. Louis River was designated an AOC due to the presence of sediment contamination, poor water quality, reduced fish and wildlife populations, and habitat loss. The river has been impacted by both legacy contaminants and current point and nonpoint source pollution. Elevated levels of sediment-associated contaminants have been documented, including polycyclic aromatic hydrocarbons (PAHs), polychlorinated biphenyls (PCBs), dioxins/furans, heavy metals and mercury (Crane and Schubauer-Berigan 1997; Crane 2006). More recently chemicals of emerging concern, such as hormones, pharmaceuticals, and personal care products have been detected (Christensen et al. 2012; Lee et al. 2012). Chemical monitoring of water and sediments indicates that contaminants (presence and concentrations) are not uniformly distributed throughout the AOC (Crane and Schubauer-Berigan 1997; Christensen et al. 2012; Lee et al. 2012). The objectives of this study were to (1) document the overall prevalence of microscopically-verified skin and liver tumors in the St. Louis River AOC for delisting purposes; (2) compare areas within the AOC in regard to prevalence of external abnormalities and liver lesions; and (3) evaluate fish health biomarkers in terms of habitat usage as indicated by stable isotope analysis.

## Materials and methods

### Site descriptions

**Fig. 1:**
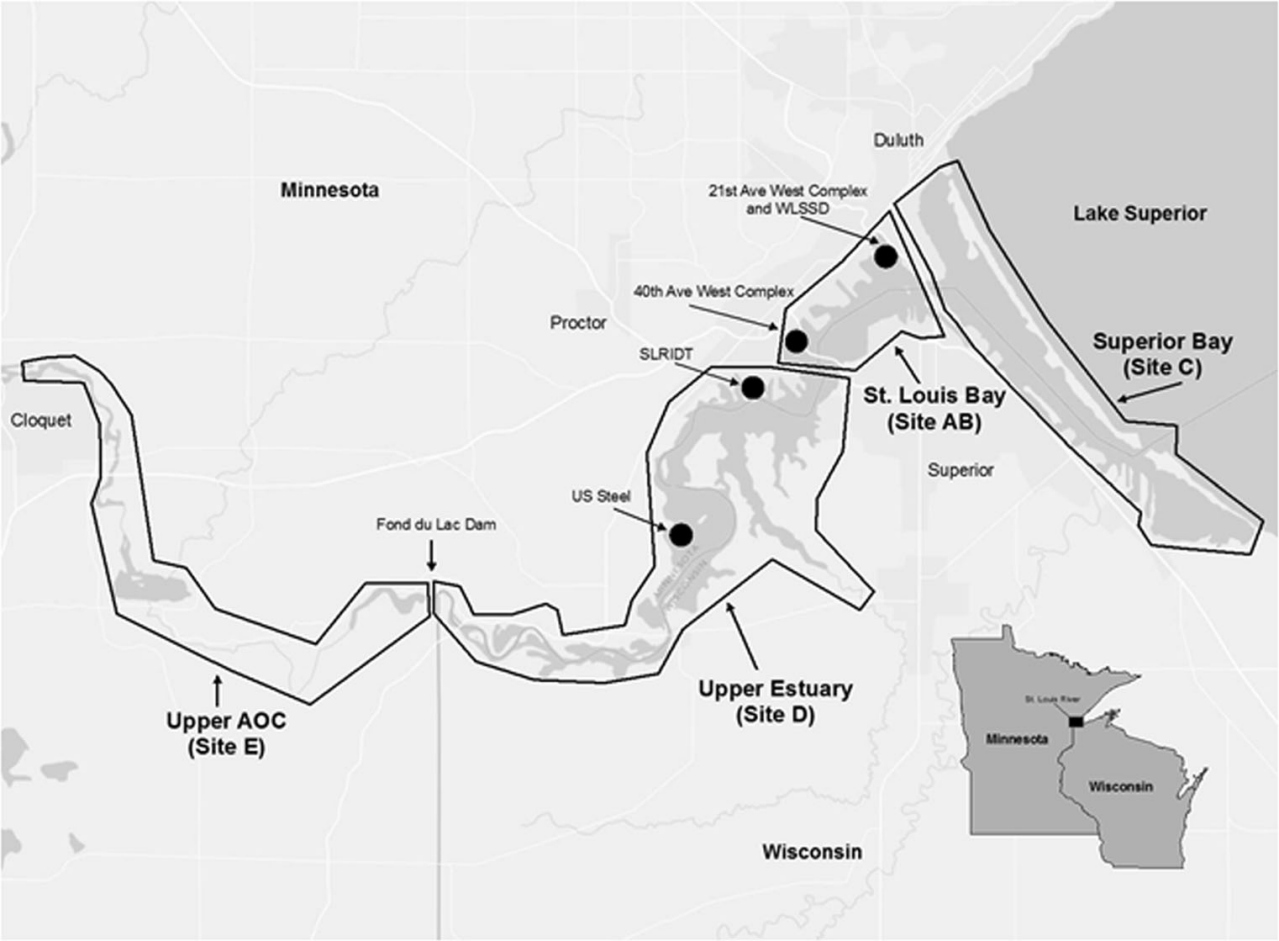
Sampling sites within the St. Louis River area of concern (AOC) included Superior Bay (C), St. Louis Bay (AB), the upper estuary (D) and the upper AOC (E)

The St. Louis River, a major U.S. tributary to Lake Superior, begins in northeastern Minnesota and flows south-southeast ultimately draining north into the western portion of Lake Superior at the border of Minnesota and Wisconsin. The St. Louis River AOC basin area is approximately 10,949.65 km^2^, making it the nation’s largest AOC. It includes the approximately 39 mainstream river miles downstream from the city of Cloquet (inclusive of the portion downstream of the Fond du Lac dam and the St. Louis estuary), Superior Bay (Fig. 1), Allouez Bay (a large embayment in the northeastern corner of the AOC), and the lower Nemadji River. The lower St. Louis River takes on the characteristics of a freshwater estuary and is affected by seiche effects that complicate the transport of chemicals within the system (Christensen et al. 2012). The AOC has numerous potential sources of contaminants, including two active Superfund sites, the St. Louis River/Interlake/Duluth Tar site (SLRIDT) and the US Steel Corporation’s former Duluth Works Site (Fig. 1).

Adult white sucker were sampled from four different areas of the AOC (Fig. 1) designated based on hydro-geomorphic setting and differing with respect to land use characteristics and differences in major sources of sediment contamination. The most downstream area of the AOC sampled was Superior Bay (site C) which is subject to a large amount of cargo vessel and recreational boat traffic. Adjacent land is currently (and was historically) highly modified by filling wetland and open waters for industrial land uses including petroleum refineries. The St. Louis Bay sampling area (site AB) was located just upriver of Superior Bay and is also subject to a large amount of cargo vessel traffic. This area is impacted by historical and current urban and industrial influences including power generating facilities, railroad yards, the Western Lake Superior Sanitary District (WLSSD) treatment facility discharge and the Erie Pier Confined Disposal Facility (for dredge materials). The Upper Estuary sampling area (site D) was located upstream of St. Louis Bay and extended to the Fond du Lac dam. This area is subjected to limited barge traffic and limited current urbanization, however it had more historical industrialization than sites AB and C, and is home to both Superfund sites. The most upstream sampling area was the Upper AOC (site E) located from above Cloquet, MN downstream to the Fond du Lac dam. This area is considered less impacted with minimal current industrialization and urbanization upstream.

### Field collections

In 2011, white sucker were collected during the spring spawning period, in each of the four areas. Twenty-five were collected at sites A (near the 21st Ave. West complex; WLSSD) and B (near the 40th Ave West complex) which were combined into one site AB (St. Louis Bay), while 50 were collected at each of sites C, D, and E (Fig. 1) by seine, trap-nets and both backpack and boat electroshocking. Individuals greater than 250 mm in length were targeted in order to ensure they were 3 years of age or older. Fish were held in nets (<24 h) in the river until processed. They were euthanized with Finquel™ (MS222; Argent Chemical Laboratories, Inc., Redmond, WA), weighed to the nearest gm and total length measured to the nearest mm. A necropsy-based assessment was completed and all external and internal abnormalities were recorded. Any grossly visible abnormalities, similar to those described by Smith et al. (2002) and Rafferty and Grazio (2006), were removed and placed in Z-Fix™ (Anatech LTD, Battle Creek, MI, USA) preservative. From all fish, five to eight pieces of liver from areas throughout the organ and a piece of gonad were removed and placed into fixative. A 1 cm × 1 cm section of dorsal muscle tissue was sampled from between the head and dorsal fin, placed in a small plastic bag and kept on ice, then frozen for stable isotope analyses. Lapillus otoliths, the most accurate structures for aging of white sucker (Sylvester and Berry 2006) were removed during necropsy and placed in a labeled coin envelope for subsequent processing and analysis.

### Laboratory analyses

Pieces of raised lesions on the body surface, fins or lips were decalcified (Cal-Ex Decalcifier, Thermo Fisher Scientific Inc., Pittsburgh, PA). Skin, liver and gonad tissues were routinely processed for histology, embedded in paraffin, sectioned at 6 μm, and stained with hematoxylin and eosin (Luna 1992). There are currently no diagnostic criteria for skin or liver tumors of white sucker. Hence, nonneoplastic, preneoplastic and neoplastic changes were documented, following previously described diagnostic criteria for liver (Boorman et al. 1997; Wolf and Wolfe 2005; Blazer et al. 2006, 2007) and skin (Blazer et al. 2007) for brown bullhead (*A. nebulosus*) and other species. For most of the biomarkers assessed (grossly visible raised skin lesions, skin and liver neoplasms, foci of cellular alteration, helminth and myxozoan parasites) presence or absence was used for statistical analyses. Macrophage aggregate density in liver was subjectively rated on a scale of 0–4 based on the number and size, similar to that described in Fournie et al. (2001).

Lapillus otoliths were prepared by a method modified from Koch and Quist (2007) using a multiple-stage process. First, the caps of plastic 2.0-mL flat-top microcentrifuge tubes (Fisher Scientific, Pittsburgh, PA, USA) were filled with modeling clay and the tapered ends removed to create a cylinder. Single lapilli were placed into the clay such that the “thumb” or anterior end of the otoliths was embedded into the clay. The vial was filled using the Epoxicure brand of resin and hardener (Buehler Inc., Lake Bluff, IL, USA) and allowed to harden. The plastic case was removed and otoliths sectioned at 0.08 mm thickness using an Isomet low speed saw (Buehler Inc., Lake Bluff, IL, USA). Annuli were counted under transmitted light using a light microscope.

Dorsal muscle tissue samples were dried (55 °C for 24 h), ground, and 0.7 mg packed into a tin capsule for stable isotope analysis. Samples were analyzed using a PDZ Europa ANCA-GSL elemental analyzer interfaced to a PDZ Europa 20-20 isotope ratio mass spectrometer (University of California-Davis Stable Isotope Facility). Stable isotope ratios are reported in δ notation in which δX:δX = (*R*_sample_/*R*_standard_ − 1) × 10^3^, where X is the C or N stable isotope, *R* is the ratio of heavy:light stable isotopes, and Vienna Pee Dee Belemnite and air are the standards for δ^13^C and δ^15^ N, respectively. The analytical error, the mean standard deviation (SD) of replicate laboratory reference material, was ±0.1 % for both δ^13^C and δ^15^N. The δ^13^C values were corrected for lipid content because the molar C:N varied among individuals (range 3.6–6.7), indicating variable lipid content; we used the mass balance correction proposed by Hoffman and Sutton (2010). A total of 198 fish were analyzed (one sample from each of sites D and E were not successfully analyzed).

To translate fish tissue stable isotope data into estimates of habitat usage, we used a C and N stable isotope mixing model to quantify how much of a white sucker’s recent (6 months to 2 years) diet was derived from each of three regions: Lake Superior, the lower estuary (Superior Bay, C and St. Louis Bay, AB), and upper estuary (site D). A proportional contribution (based on “source” isotopic signatures) to the fish’s isotopic signature from each of the three geographic regions, or “sources” was calculated using the model, assuming mass balance (i.e. the proportions must add to 1; Phillips and Gregg 2001). As such, a fish with an isotopic signature that is exactly intermediate between the three sources would have a contribution of 0.33 from each region (i.e. it acquired one-third, or 33 % of its recent diet from each region). We ran a mixing model for each white sucker sampled below Fond du Lac dam (*n* = 149). Individual fish δ^13^C and δ^15^N values were fit to the model by constraining the model results (proportions) to between 0 and 1. We preferentially fit δ^15^N values because small deviations in trophic level have a much larger effect on the fish’s δ^15^N value than δ^13^C value. Where required, the mean absolute fit value for δ^15^N was 0.7 % (SD = 0.7 %, *n* = 65), and for δ^13^C, it was 1.5 % (SD = 0.7 %, *n* = 4). Using error propagation, a standard deviation was calculated for the contribution from each habitat for each individual (Phillips and Gregg 2001).

We used both captured white sucker and available data on benthic resources to define the “sources” for the mixing model because similar data were not available for each study site. For upper estuary (site D) fish the isotopic signature was defined as the average δ^13^C and δ^15^N of fish captured at site E (δ^13^C_upper estuary_ = −30.7 %, δ^15^N_upper estuary_ = 10.2 %). We used the upper AOC (site E) because Fond du Lac dam restricts downstream movement (hence, migration between site E and the Lake is not possible) and because these two sites have similar geomorphological (riverine) and biogeochemical characteristics. For lower estuary fish (Superior and St. Louis Bays, sites C and AB) the mean δ^13^C of larval midges (Chironomidae) and mayflies (Ephemoptera) captured in the lower estuary (−23.9 %, SD 2.7 %) as previously reported by Hoffman et al. (2010) was used, correcting for one trophic level fractionation (+0.4 %, i.e. δ^13^C_lower estuary_ = −23.5 %). Whereas, benthic invertebrate prey δ^13^C values are similar from Fond du Luc dam to St. Louis Bay and are increasingly higher from St. Louis Bay to Lake Superior, δ^15^N values of benthic invertebrates are similar throughout the estuary (Hoffman et al. 2010). Thus, the same δ^15^N values were used for the upper and lower estuary sites (δ^15^N_lower estuary_ = 10.2 %). For Lake Superior fish, the isotopic signature was based on the mean value of amphipods, isopods and decapods in shallow, inshore waters (−16.4 % δ^13^C, 0.5 % δ^15^N; Strand 2005), correcting again for trophic fractionation (+0.4 % δ^13^C, +3.4 % δ^15^N). The corrected δ^15^N, however, was about 1 % less than any observed white sucker, and so it was revised upward for the model; δ^13^C_Lake Superior_ = −16.0 % and δ^15^N_Lake Superior_ = 5 %.

### Statistical analyses

Data were checked for normality and equality of variance and if normally distributed were compared using Tukey’s multiple comparison test (SYSTAT 11.0, Systat Software Inc., Chicago, IL, USA). Data not normally distributed were compared using nonparametric ANOVA followed by Dunn’s multiple comparison test to compare site medians with GraphPad InStat 3 software (GraphPad Software, Inc., La Jolla, CA, USA). Logistic regression was used to explore potential relationships between habitat usage (i.e. amount of diet derived from a certain area based on stable isotope signatures) and fish health indicators. The health variables included grossly visible raised skin lesions, skin neoplasms, presence of a bile duct myxozoan, presence of helminth parasites in liver tissue, foci of cellular alteration in the liver, liver neoplasms and macrophage aggregates in the liver. The four factors considered in the logistic regression were age, sex, % upper estuary contributing to diet and % lake contributing to the diet. The upper estuary variable was included because this is the portion of the system in which two Superfund sites are located (Fig. 1). The lake variable was included because a lake versus estuary distinction might be significant given the many sources of contamination to the AOC. An α-level of 0.05 was used to indicate significance in all tests.

## Results

### Morphometric measurements

**Fig. 2:**
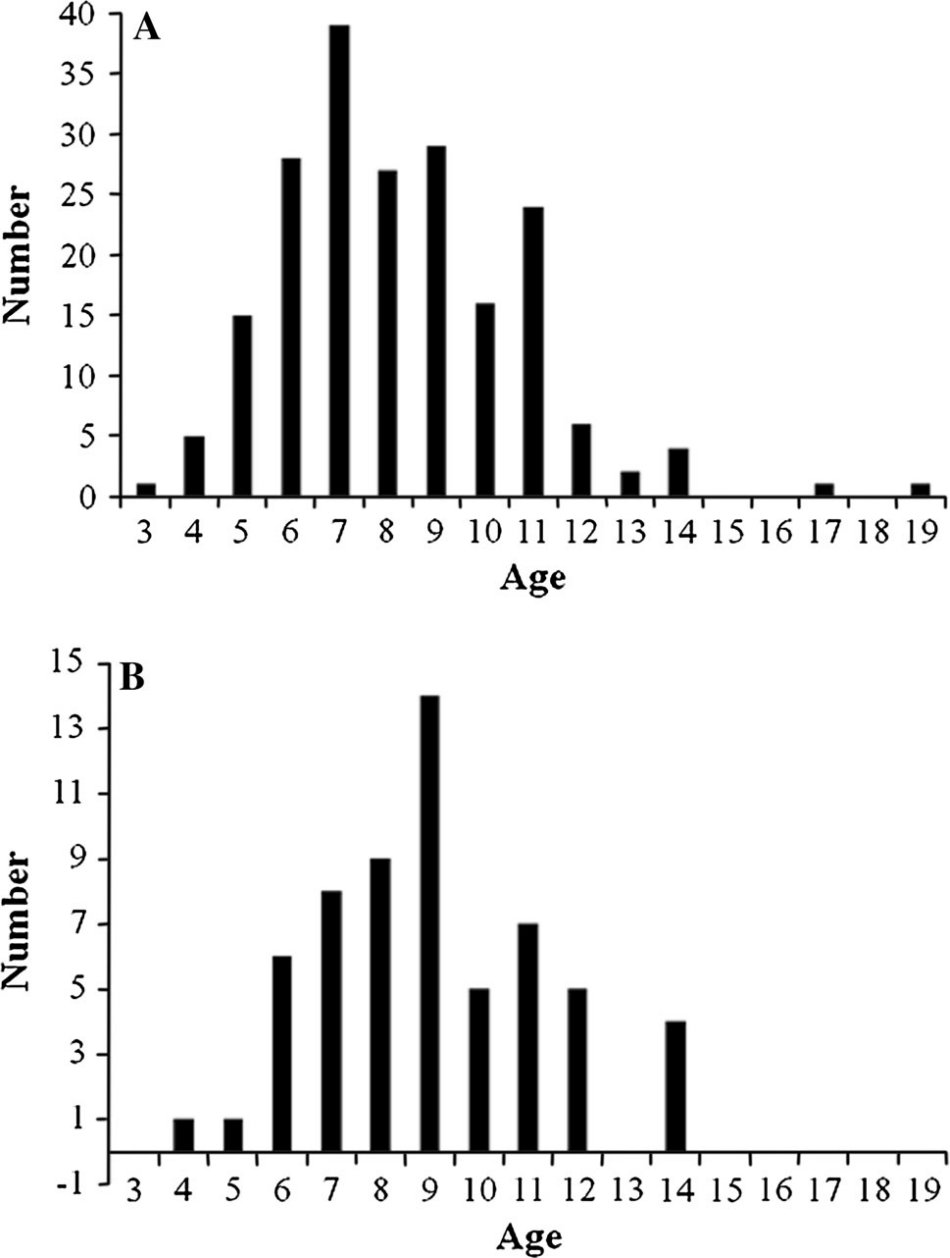
**a** Age distribution of white sucker collected throughout the St. Louis River area of concern. **b** Age distribution of white sucker with grossly visible raised skin or lip lesions

**Fig. 3:**
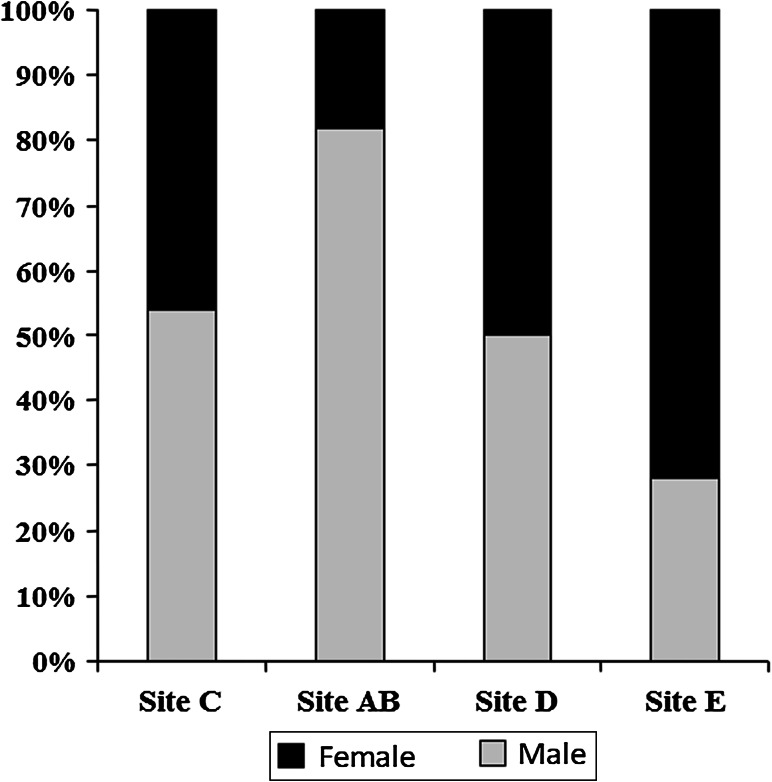
Sex ratios observed in white sucker collected at four sites: Superior Bay (C), St. Louis Bay (AB), upper estuary (D), upper AOC (E) within the St. Louis area of concern (AOC)

A total of 200 white sucker (94 female and 106 male) were collected from four areas within the St. Louis River AOC. They ranged in age from 3 to 19 year, with the majority in the 6–11 year range (Fig. 2a). There was a difference (Kruskal–Wallis ANOVA, KW = 14.27, *p* = 0.0026) among the sites in mean age. White sucker collected within St. Louis Bay (AB) and the upper estuary (D) were older than those from Superior Bay (C) and fish from the upper AOC (E) were intermediate. White sucker from site C were also shorter (Kruskal–Wallis ANOVA, KW = 32.54, *p* < 0.0001) and lighter (Kruskal–Wallis ANOVA, KW = 37.56, *p* < 0.0001) than those from D and E but not different than AB (Table 1). There was a difference in the sex ratio among the sites. Sites C (Superior Bay area) and site D (upper estuary) had approximately even numbers of male and female. Only 18 % of the suckers collected at site AB were female while 72 % of those collected at site E were female (Fig. 3).

**Table 1:** Morphometric characteristics and stable isotope ratios of white sucker collected Spring 2011 within the St. Louis area of concern (AOC)

Site	Sample size^A^	Age^B^ (years)	Length (mm)	Weight (gm)	δ^13^C (%)	δ^15^N (%)
Site C^C^	50	7.3 ± 2.3^a^	395.8 ± 65.1^a^	678.9 ± 209.1^a^	−23.2 ± 3.0^a^	9.0 ± 1.4^a^
Site AB^C^	50	8.6 ± 2.5^b^	412.2 ± 36.5^ab^	756.9 ± 212.8^a^	−23.5 ± 2.9^a^	8.6 ± 1.7^a^
Site D^C^	50 (49)	8.8 ± 2.8^b^	430.0 ± 42.5^b^	959.6 ± 334.6^b^	−23.8 ± 3.6^a^	8.5 ± 1.5^a^
Site E^C^	50 (49)	8.1 ± 1.9^ab^	430.8 ± 36.2^b^	900.9 ± 231.5^b^	30.7 ± 1.4^b^	10.2 ± 1.0^b^

^A^Number in parentheses are sample sizes for stable isotope ratios

^B^Data presented as mean ± SD. Values within a column followed by the same letter are not significantly different

^C^Site C = Superior Bay, site AB = St. Louis Bay, site D = upper estuary and site E = upper AOC

### Gross observations

**Fig. 4:**
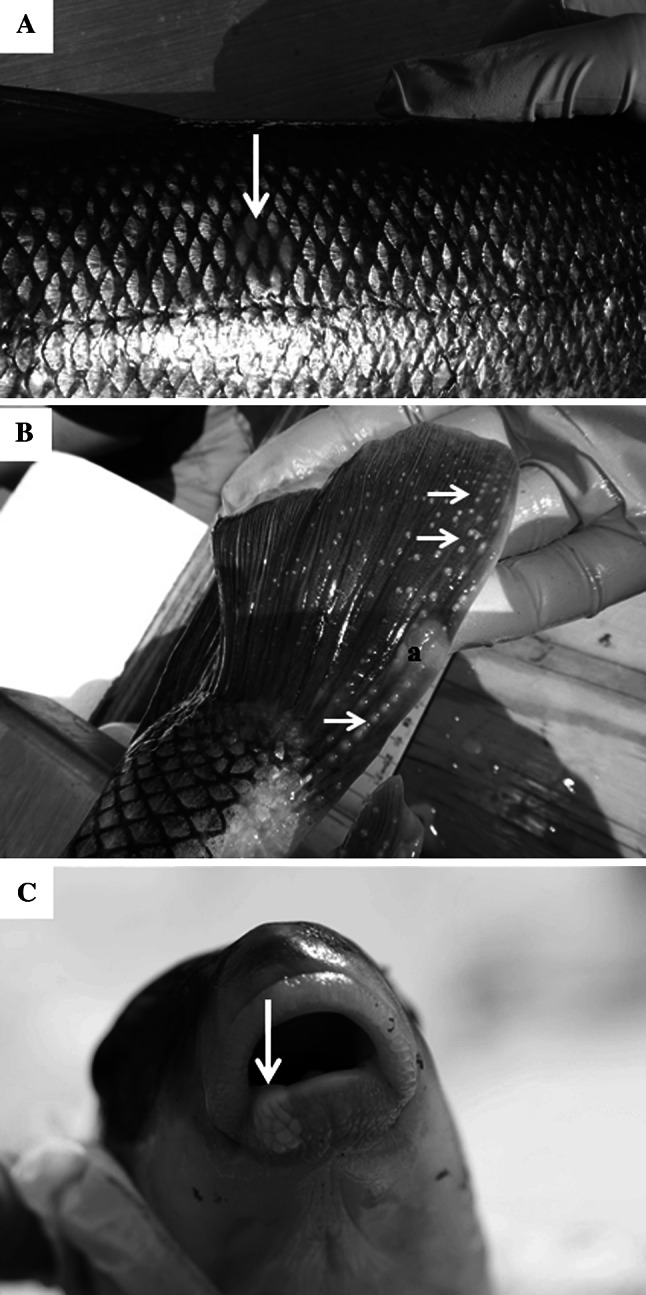
Skin lesions of white sucker collected within the St. Louis area of concern. **a** Slightly raised mucoid lesion (*arrow*) on the body surface. **b** Raised, white to creamy lesion (*a*) on the caudal fin of a male white sucker with breeding tubercles (*arrows*). **c** Slightly raised lip lesion (*arrow*)

A variety of grossly visible lesions were noted including red/eroded skin lesions, melanistic areas, abnormal, missing or opaque eyes, abnormal-appearing breeding tubercles, and raised lesions. Raised lesions included slightly raised, pale/creamy areas on fin or body surface, slightly raised, smooth mucoid plaques on the body surface and smooth to rough lip lesions (Fig. 4). There was one lip lesion observed in suckers from St. Louis Bay (AB) and two from the upper estuary (D). One melanistic area was observed on a white sucker from Superior Bay (C) and two from the upper AOC (E). Two abnormal eyes were noted in suckers from site AB, three from site C, two from site D and two from site E.

### Microscopic observation: skin

**Fig. 5:**
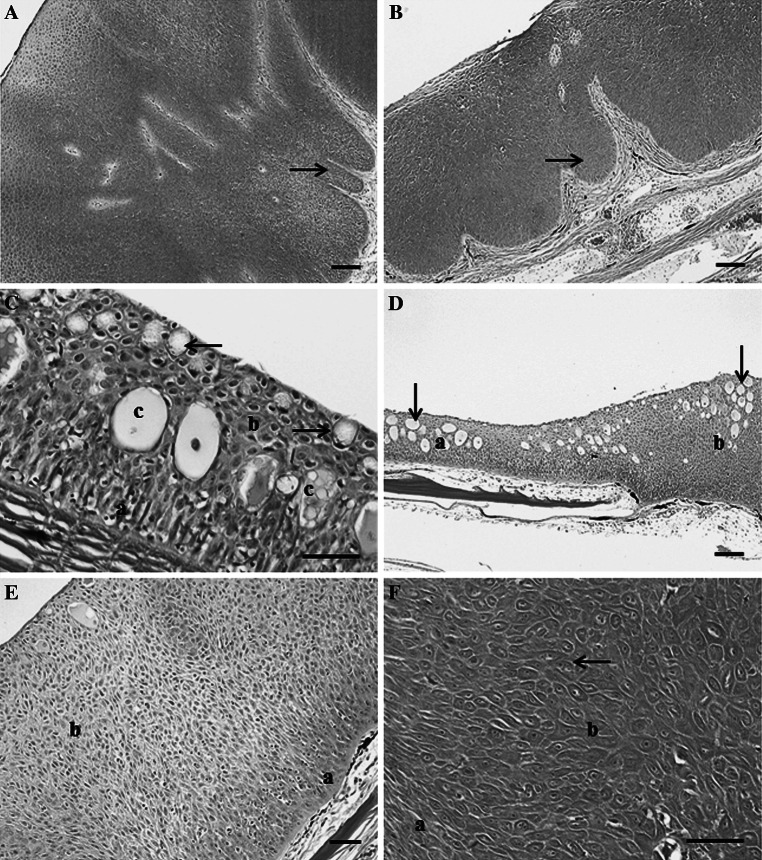
Microscopic pathology observed in white sucker collected within the St. Louis River area of concern. **a** Lip papilloma composed of proliferating epithelial cells (*a*) within the epidermis, leading to folds of tissue extending into the underlying layers (*arrow*). *Scale bar* 100 μm. **b** Body surface papilloma with folds of proliferating cells (*arrow*). *Scale bar* 100 μm. **c** Normal epidermis of white sucker skin contains a layer of columnar cells (*a*) along the basement membrane, flattened, squamous epithelial cells (*b*) toward the superficial surface with club cells (*c*) and goblet cells (*arrows*) present. *Scale bar* 50 μm. **d** Section of skin containing normal epidermis (*a*) and an area of hyperplastic epidermis (*b*). Epithelial and fright/alarm cells (*arrows*) are present in both areas. *Scale bar* 100 μm. **e** Hyperplastic lesions contained a basal layer of columnar cells (*a*) and hyperplasia epithelial cells (*b*). *Scale bar* 50 μm. **f** Cell within the papillomas vary in size and shape from thin elongated cells (*a*) to larger, plumper cells (*b*) which in some cases were binucleated (*arrow*). *Scale bar* 50 μm. Hematoxylin and eosin stain

The three lip lesions were all papillomas (Fig. 5a), as were six of the raised fin/body surface lesions (Fig. 5b). These lesions were composed of proliferating epithelial cells forming a thickened epidermis and epidermal pegs extending into the dermis. However, invasion of cells through the basement membrane and into the dermis was not observed. Six of the nine skin neoplasms were observed in white sucker collected from St. Louis Bay (site AB), two from site D and one from site E (Table 2). The remaining raised lesions were hyperplastic lesions. Normal white sucker skin is composed of epidermis of varying thickness with a basal layer of columnar cells, cuboidal cells in the middle layer and flattened superficial cells. Goblet (mucous) and club cells are also present in the epidermis (Fig. 5c). The hyperplastic lesions contained proliferations of both club and epithelial cells (Fig. 5d) or primarily epithelial cells (Fig. 5e). In some cases, these areas lacked the presence of goblet and club cells and there was abnormal arrangement of the middle layers of epidermis, although cell size and appearance of epithelial cells was generally normal. Epithelial cells within the papillomas varied in size and shape, and occasionally multinucleated cells were observed (Fig. 5f).

**Table 2:** Percentage of white sucker with specific abnormalities collected within the St. Louis River area of concern (AOC)

Abnormality	Superior Bay (C)	St. Louis Bay (AB)	Upper estuary (D)	Upper AOC (E)	Total (%)
Raised skin lesions					
Body surface/fins	10	42	28	38	29.0
Lips	0	2	4	0	1.5
Total	10	44	32	38	**31.0**
Skin neoplasia					
Papilloma	0	12	4	2	**4.5**
Liver					
Altered foci	8	6	4	0	**4.5**
Bile duct proliferation	52	40	58	46	**49.0**
Cholangioma	2	2	2	0	1.5
Cholangiocarcinoma	2	2	4	4	3.0
Total liver neoplasms	4	4	6	4	**4.5**

Sample size for each site was 50

Bold values indicate totals for each major category

### Microscopic observations: liver

**Fig. 6:**
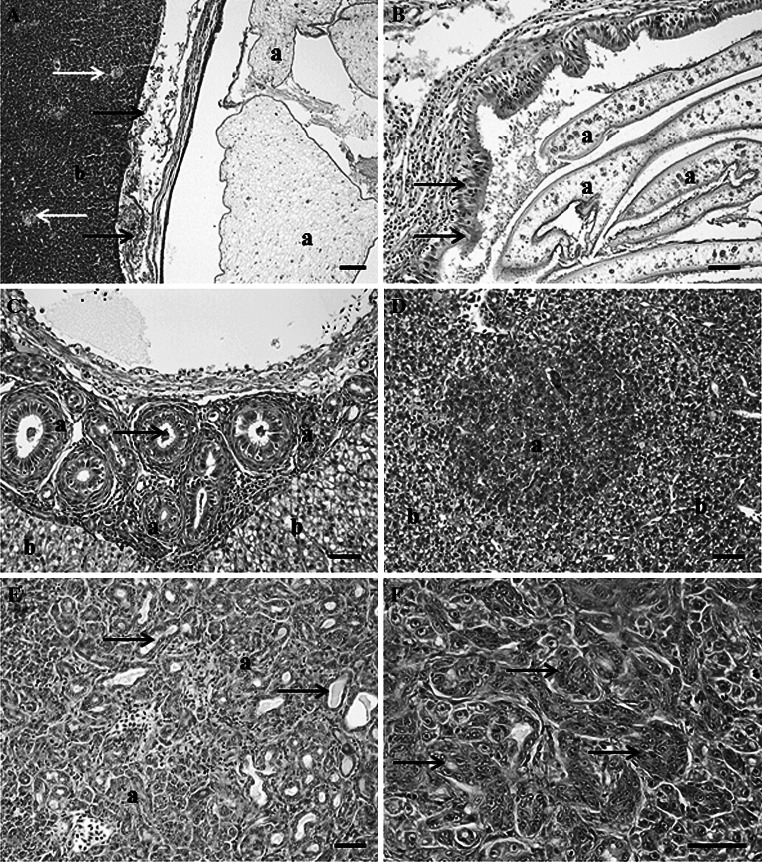
Microscopic pathology observed in the liver of white sucker collected within the St. Louis area of concern. **a** Encysted cestode (*a*) within the liver tissue (*b*) separated from hepatocytes by a layer of inflammatory and necrotic cells (*black arrow*). Macrophage aggregates (*white arrows*) were commonly observed within hepatic tissue. *Scale bar* 100 μm. **b** Sporoplasm of a myxozoan parasite (*a*) within a distended bile duct with areas of epithelial hyperplasia (*black arrows*). *Scale bar* 50 μm. **c** Focal area of bile duct proliferation (*a*) within hepatic tissue (*b*) with cross-sections of myxozoan sporoplasms (*arrows*). *Scale bar* 50 μm. **d** Foci of cellular alteration (*a*) within hepatic tissue (*b*). *Scale bar* 50 μm. **e** Section of a cholangiocarcinoma (*a*) illustrating well differentiated proliferating bile ductules containing bile (*arrows*). *Scale bar* pleomorphic cells, 50 μm. Hematoxylin and eosin stain

There were numerous non-neoplastic microscopic lesions observed within the liver including parasite-induced inflammatory and proliferative changes. Large cysts of helminth parasites were present in and on the liver surface of white sucker captured below Fond du Lac Dam (Fig. 6a). They were not observed in fish captured at site E. These cysts caused primarily pressure necrosis and granulomatous inflammation. In addition, there were plasmodia, often large, of a myxozoan parasite in the bile ducts of white suckers captured at all sites (Fig. 6b). Bile duct proliferation, fibrosis and sometimes inflammation were observed (Fig. 6c), in association with the myxozoan parasite. Foci of cellular alteration (FCA) were only observed at the sites below the dam (Table 2) and these included vacuolated and basophilic foci (Fig. 6d). There was a low prevalence (4.5 %) of liver neoplasms, all of which were of bile duct origin. These were distributed throughout all four sites with no difference among the sites (Table 2). Grossly, some of these lesions were noted as slightly raised areas with a green discoloration. Microscopically, proliferating bile ducts with bile stasis was observed (Fig. 6e). A total of eight white sucker had bile duct neoplasms, six of them were cholangiocarcinomas (Fig. 6f). Age was only available for seven of these samples as no otoliths were collected from one. Density (mean ± SD) of macrophage aggregates (Fig. 6a) ranged from moderate to extensive. Density scores of suckers from Superior Bay (1.5 ± 1.0) were significantly lower (Kruskal–Wallis ANOVA, KW = 25.57, *p* < 0.0001) than those from St. Louis Bay (2.6 ± 1.3) and the upper estuary (2.5 ± 1.2), while those from the upper AOC (E) were intermediate (2.1 ± 1.4). This is the same pattern observed for fish age and studies in other fish species have shown MA to increase with age (Brown and George 2006).

### Stable isotope composition of fish tissue

**Fig. 7:**
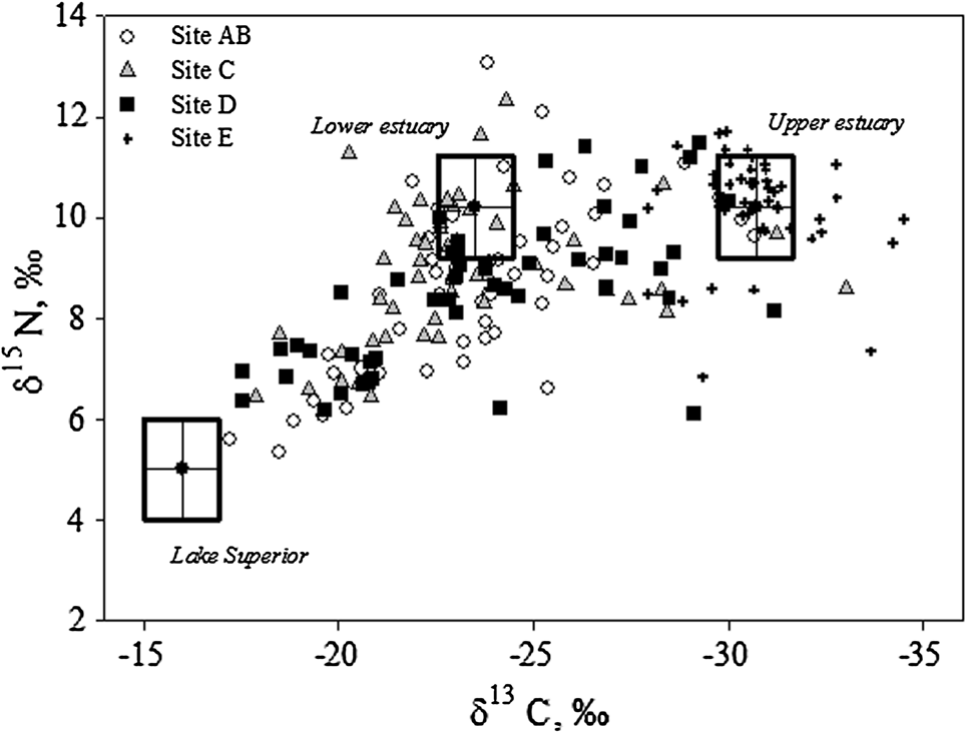
Distribution of δ^13^C and δ^15^N values in muscle tissue of white sucker collected at four sites Superior Bay (C), St Louis Bay (AB), upper estuary (D), and upper AOC (E) within the St. Louis area of concern (AOC). The lower estuary includes sites C and AB

The δ^13^C and δ^15^N values of fish tissue encompassed the range of values expected for fish moving between Lake Superior and the upper estuary (Fig. 7). No fish had a value that exactly matched the Lake Superior signature (δ^13^C_Lake Superior_ = −16.0 %, δ^15^N_Lake Superior_ = 5 %), whereas we did capture individuals that matched either the lower estuary (St. Louis Bay and Superior Bay; δ^13^C_lower estuary_ = −23.5 %, δ^15^N_lower estuary_ = 10.2 %) or upper estuary signature (δ^13^C_upper estuary_ = −30.7 %, δ^15^N_upper estuary_ = 10.2 %). Most individuals had a signature that suggested they had been feeding between Lake Superior and the lower estuary, or else between the lower and upper estuary. A few fish had δ^15^N values <8 % and δ^13^C values between about −32 to −25 %; these are interpreted to have fed mostly between Lake Superior and the upper estuary. Four individuals had unusually high δ^15^N values, >11 %.

Within each of the estuary sites, fish exhibited the full range of possible isotopic signatures, indicating that there was no relationship between fish migration history and the capture location. In contrast, fish from the upper AOC (E), which are physically isolated from the estuary by the Fond du Lac dam, were isotopically distinct. Accordingly, estuarine sites were significantly different from site E but similar to one another (Table 1; δ^13^C ANOVA: *F* = 77.6, *df* = 3, *p* < 0.001; δ^15^N ANOVA: *F* = 14.9, *df* = 3, *p* < 0.001).

**Fig. 8:**
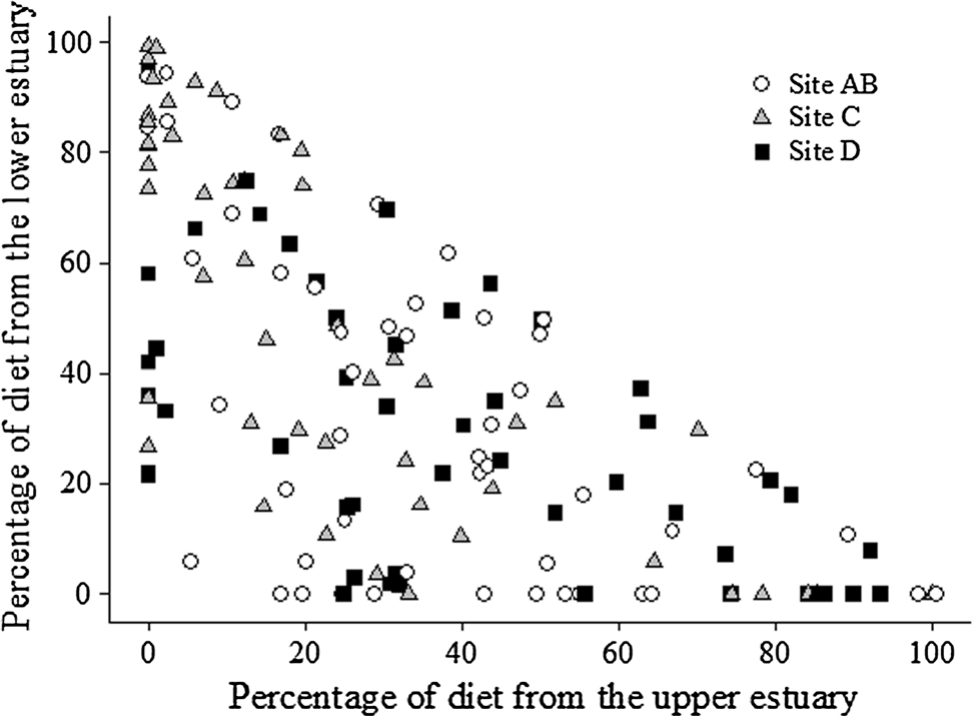
Results of the dual stable isotope mixing model for white sucker captured below Fond du Lac Dam. The data points represent the dietary contribution to each fish from the upper estuary (site D) and lower estuary (Superior Bay C, St. Louis Bay AB, combined). The sum of all three sources (upper estuary, lower estuary, Lake Superior) total 100 %; thus, for any point, the Lake Superior contribution is the remainder of the sum of the upper and lower estuary contributions. That is, the points in the lower left corner of the plot represent fish with a high dietary contribution from Lake Superior. The *shading* indicates the capture location of the fish (*gray* site C, *open* site AB, *closed* site D)

The results of the stable isotope mixing model reflect the distribution of stable isotope signatures (Fig. 8). Most fish captured below Fond du Lac dam relied on the estuary for some portion of their diet; only seven of the 149 fish analyzed had a >75 % contribution of Lake Superior to its diet. In contrast, 22 fish derived >75 % of their diet from the lower estuary and 17 fish derived >75 % of their diet from the upper estuary. The remainder of white sucker (103 individuals), relied on a mix of locations for feeding; 68 of those derived 25–75 % of their diet from Lake Superior, 61 derived 25–75 % of their diet from the lower estuary, and 66 derived 25–75 % of their diet from the upper estuary.

The standard deviations (SD) associated with the contribution estimates were source-dependent. For the upper estuary contributions, the mean SD was 0.42 for contributions <5 %, and declined to 0.22 for contributions >95 %. For the lower estuary contributions, the SD was similar over the range of contributions; the mean was 0.32 for contributions <5 % and 0.29 for contributions >95 %. For the Lake Superior contributions, the mean SD was 0.17 for contributions <5 % and increased to 0.38 for contributions >80 % (no contributions over 95 % were estimated).

### Logistic regression analysis

The power of the skin and liver neoplasms logistic regression models was impacted by the low incidence rate (Table 3). For fish captured at sites A–D (below Fond du Lac dam), the incidence of grossly observable skin lesions significantly (*p* < 0.05) increased with the proportion of the fish’s diet that was obtained from the upper estuary, and also increased with age, though the latter was weakly supported (*p* < 0.10). Skin neoplasm incidence also increased with feeding in the upper estuary, though the factor was weakly supported (*p* < 0.10); neither age nor sex was a significant factor. The incidence of liver neoplasms, which were all bile duct tumors, was not significantly related to any factor. In contrast, FCA incidence significantly increased with age, as did the density of MA. The incidence of myxozoan parasites, as observed through histologic sections, was significantly lower in females than males.

**Table 3:** Logit coefficients from logistic regression models for biological abnormalities, multiple linear regression for macrophage aggregates (MA)

Factors	Raised skin	Neoplastic skin	Myxozoan	Helminth	Altered foci	Liver neoplasia	MA
Constant	**−2.18**	**−4.28**	**−1.40**	**2.76**	**−5.41**	**−4.88**	**0.82**
Sex	−0.46	−14.15	**−0.99**	0.08	0.09	−0.33	
Age	*0.13*	−0.04	0.09	−0.09	**0.31**	0.11	**0.16**
Upper estuary	**1.55**	*3.34*	−0.49	−*1.39*	−3.40	1.96	−0.07
Lake	−0.72	2.97	0.42	**−2.18**	1.16	0.40	0.10

An italicized value indicates a *p* value between 0.05 and 0.10, while a bold value had a *p* value < 0.05

Since both raised skin lesions and skin neoplasms increased with the proportion of diet obtained from the upper estuary, the associated odds ratios were estimated. We binned the fish into four age groups (ages 3–6; 7–8; 9–10; 11+) of approximately equal size to account for a possible age effect, as indicated by the logistic regression analysis (Table 4). For both skin lesions and neoplasms there was an age effect. For fish 9 years or older (*n* = 63), the proportion of fish with a skin lesion or neoplasm present was 0.33 and 0.10, respectively, whereas it was 0.25 and 0.02 for fish less than age 9 (*n* = 84, Table 4). Based on the stable isotope results, fish with either skin lesions or neoplasms fed more in the upper estuary (>43 %) and less in the lake (<36 %) than those with no skin lesions (<32 % upper estuary, >40 % lake, Table 5). A similar habitat pattern was observed in white sucker with liver neoplasms (Table 5). Based on this result, fish were binned by habitat use into three groups: fish captured at sites A–D that obtained >50 % of their diet from the upper estuary, fish that obtained the majority of their diet somewhere else (<50 % upper estuary), and fish captured at site E (which fed within site E). Age 9+ white sucker that obtained >50 % of their diet from the upper estuary were 1.7 times more likely to exhibit a skin neoplasm than suckers that obtained <50 % of their diet from the upper estuary, and 2.9 times more likely than suckers captured above Fond du Lac dam (site E). Suckers that obtained >50 % of their diet from the upper estuary were 2.2 times more likely to exhibit a liver neoplasm than those that obtained <50 % of their diet from the upper estuary, but 1.3 times more likely than those captured in the upper AOC (site E) (Table 6).

**Table 4:** Number of white sucker from the St. Louis area of concern below Fond du Lac Dam with raised skin lesions and neoplasms based on four age groupings

Age range	Total	Raised lesions	Skin neoplasms	Liver neoplasms
3–6	40	8	1	3
7–8	44	13	1	1
9–10	31	9	3	0
11+	32	12	3	2

This includes Superior Bay (site C), St. Louis Bay (site AB) and the upper estuary (site D)

**Table 5:** Habitat effect on presence of raised skin lesions, skin neoplasms and liver neoplasms of white sucker captured in the St. Louis River area of concern below Fond du Lac dam

Group	Total	% Upper estuary^a^	% Lower estuary^a^	% Lake
Raised lesions	21	**45**	26	29
No raised lesions	42	26	28	**45**
Skin neoplasm	6	**43**	21	36
No neoplasm	57	32	28	**40**
Liver neoplasm	6	**47**	23	30
No neoplasm	141	33	37	**30**

For skin lesions and skin neoplasms only age 9+ are included, for liver neoplasms all white sucker are included

Bold values indicate the habitat with the greatest effect on each response

^a^Upper Estuary is site D, Lower Estuary includes Superior Bay (C) and St. Louis Bay (AB)

**Table 6:** Habitat-based odds ratio for skin (age 9+ fish only) and liver (all ages) neoplasms for white sucker captured in the St. Louis area of concern (AOC)

Neoplasms	Site	Diet	Absent	Present	Odds
Skin	A–D	>50 % upper estuary	13	2	0.15
	A–D	<50 % upper estuary	44	4	0.09
	E	100 % site E	19	1	0.05
Liver	A–D	>50 % upper estuary	36	3	0.08
	A–D	<50 % upper estuary	106	4	0.04
	E	100 % site E	47	3	0.06

Site AB St. Louis Bay, C Superior Bay, D upper estuary and E upper AOC (above Fond du Lac dam)

## Discussion

A high percentage (31 %) of white sucker collected within the St. Louis AOC had grossly observable raised skin and lip lesions, however, the prevalence of either skin or liver neoplasms was low. Despite the low incidence, the amount of a white sucker’s diet that was obtained from the upper estuary was a significant predictor of skin neoplasm incidence. A similar habitat use pattern was observed among fish with liver neoplasms, although it was not significant. This suggests that habitat use (as indicated by stable isotopes) within the AOC may be an important risk factor and merits further investigation. The liver neoplasms, observed in 4.5 % of the suckers sampled were of bile duct origin. These were observed at all four sites with no significant difference among sites. Bile duct proliferation was also common in the liver of white sucker from all the sites. Foci of cellular alteration (FCA) were only observed at the sites below the dam. There is substantial evidence, in numerous fish species, that FCA are associated with contaminant exposure, particularly to PAHs, PCBs, DDTs, chlordanes, dieldrin, mercury, pulp mill effluent and aromatic hydrocarbon metabolites and may be preneoplastic lesions (Vogelbein et al. 1990; Murchelano and Wolke 1991; Myers et al. 1991; Myers et al. 1994; Vethaak and Wester 1996; Stentiford et al. 2003; Au 2004; Feist et al. 2004; Köhler 2004). In addition, laboratory exposures of a number of fish species, to a variety of compounds, have suggested FCA, particularly the basophilic foci, are preneoplastic (Hendricks et al. 1984; Grizzle and Thiyagarajah 1988; Hawkins et al. 1988, Hinton et al. 1988, Law et al. 1994). It is not currently known if any type (eosinophilic, vacuolated, basophilic or clear cell) progress to hepatocellular neoplasms in white sucker. Exposure to PAHs has been associated with liver carcinogenesis in a number of fish species (Baumann and Harshbarger 1995; Myers et al. 1991; Vogelbein et al. 1990). However, the role of other factors, such as estrogenic contaminants, other chemicals or the presence of a bile duct parasite initiating inflammatory and proliferative responses, needs to be explored.

The raised skin lesions were primarily mucoid plaques that by microscopic examination were hyperplastic lesions, while the three raised lip lesions observed were papillomas. Overall, 4.5 % of white sucker had skin neoplasms and all of these were papillomas. None of the raised lesions noted at Superior Bay were neoplastic, while 12 % of the white sucker collected from St. Louis Bay exhibited neoplastic skin/lip lesions. The causes or risk factors associated with skin tumors in white sucker are also still to be determined. A viral etiology has been suggested, however numerous investigators have attempted to isolate or visualize viral particles with no success (Smith et al. 1989; Premdas and Metcalfe 1996). Correlations between papilloma prevalence and persistent chemicals such as PCBs and organochlorines have been demonstrated (Premdas et al. 1995) and experimental exposures to androgens or 17β-estradiol are reported to increase papillomas in white sucker (Premdas et al. 2001). In other species, such as roach *Rutilus rutilis*, exposure to industrial and sewage effluent (Kortet et al. 2002; Korkea-aho et al. 2006, 2008) and androgens (Kortet et al. 2003) have resulted in increased prevalence and growth of papillomas.

The observations in this study as well as unpublished data from other AOCs supports the hypothesis that the mucoid plaques may be preneoplastic and further exposure may be required to “promote” carcinogenesis. The chemical or biological factors, environmental exposure levels and time necessary to promote development of neoplastic lesions from the hyperplastic lesions (mucoid plaques), assuming this occurs, are currently unknown. It is interesting to note that six of the nine white sucker with skin neoplasms were collected in St. Louis Bay which receives effluent from the WLSSD treatment facility. Isotopically, these fish did not appear to be resident in the lower estuary (St. Louis Bay). However, progression of the preneoplastic (hyperplastic) lesions to neoplasms may require less time than is necessary to modify the isotopic signature (1–2 years). The WLSSD facility collects and treats both municipal and industrial wastes of the entire region from Cloquet to Duluth. Recent monitoring of contaminants of emerging concern has shown this area to have higher levels of estrogenic contaminants, when compared to the other collection sites within the St. Louis AOC (Lee et al. 2012). Additionally, previous sediment chemical analyses and toxicity assessments found Duluth Harbor (St. Louis Bay) was generally more contaminated than Superior Bay. Hotspots of contaminated sediment occur with the most contaminated sediments in the upper estuary in the vicinity of the superfund sites (Crane and Schubauer-Berigan 1997). More recent analyses of sediment PAH concentrations found selected sites within the upper estuary, St. Louis Bay and Superior Bay exceeded sediment quality targets (Christensen et al. 2012).

Fish were collected in the spring in order to examine tumor prevalence as well as the reproductive health of fish residing with the St. Louis River AOC. However, a concern in sampling during this time period and in using a migratory species, was whether the biological responses documented can be related to the environmental conditions within the AOC. White sucker spawn in the spring over coarse substrate, usually gravel, in shallow, fast-moving water. They have been observed to travel variable distances for spawning and migration is likely dependent on finding suitable habitat (Rainey and Webster 1942; Quinn and Ross 1985; Doherty et al. 2010). Males generally arrive at the spawning grounds first and aggregate in shallow areas (Page and Johnston 1990). This pattern could account for biased sex ratio at certain sites, most notably the high percentage of males captured in St. Louis Bay just below the primary spawning grounds, which are located in the riverine portion of the estuary below Fond du Lac dam. Using a geographically widespread set of sampling locations likely avoided age and sex composition biases in the sampled population due to spawning behavior that would be encountered by sampling in a single location. The observation that the stable isotope signatures of white sucker varied widely at each site below Fond du Lac dam suggests that within a sampling site the fish are well mixed with respect to habitat use. Hence, it does not appear to be the case that the capture location of a white sucker during the spawning season was related to recent habitat use. Thus, similar to spawning migration movements of other catastomids (Grabowski and Isely 2006), suckers from both distant and nearby riverine and estuarine habitats appear to have simultaneously moved into the lower St. Louis River during the spawning migration, thereby mixing with each other.

Previously, migration patterns of fish were traced in the St. Louis AOC by exploiting naturally occurring differences in the stable isotope composition among different locations within the AOC. The food web in the upper portion of the estuary is depleted in ^13^C (i.e. lower δ^13^C values) and is progressively enriched in ^13^C (i.e. higher δ^13^C values) with increasing proximity to Lake Superior and this change is reflected in resident fishes (Hoffman et al. 2010). In contrast, the estuarine food web is enriched in ^15^N due to the presence of wastewater effluent, whereas the Lake Superior food web is depleted in ^15^N (Hoffman et al. 2012). Thus, a fish that had been feeding in Lake Superior over the past 1–2 years will have an isotopic signature that is ^13^C-enriched and ^15^N-depleted and a fish feeding in the upper estuary (Fond du Lac dam down river to St. Louis Bay) will have an isotopic signature that is ^13^C-depleted and ^15^N-enriched. A fish feeding in the lower estuary (St. Louis Bay downstream to Lake Superior) will have an isotopic signature that is intermediate in ^13^C and ^15^N abundance. The results for the white sucker sampled in this study indicate that most individuals are utilizing the estuary from below the dam to Superior Bay for a significant portion of their diet. Only seven of the 149 individuals sampled in these areas had a >75 % contribution of Lake Superior to their diet. Hence, the biological effects observed should be indicative of environmental conditions within the estuary and river, not the lake.

However, the stable isotope data do indicate that habitat use varied widely among individuals captured below Fond du Lac dam and individuals with different habitat use were captured across the estuary (i.e. there was no significant difference in stable isotope values among sites AB–D). This implies some accounting for migratory behavior may be necessary to compare specific areas within the AOC and seasonal comparisons may be important. For the stable isotope mixing model, the error in assigning fish to three different habitats is related to the isotopic separation among them (Phillips and Gregg 2001). Thus, there is a greater certainty assigning fish to a diet predominantly derived from the lake or upper estuary, than between the lake and lower estuary, or lower estuary and upper estuary. In the mixing model, estimates of a large (>95 %) contribution from the upper estuary or negligible contribution from the lake (<5 %) had the lowest SD. Thus the isotopic signature most relevant to tumor incidence, the upper estuary signature, also had relatively low SD in the model.

Besides model properties, there are a number of other considerations that are relevant to interpreting the stable isotope data. First, given the slow isotopic turnover in adult fishes (6 months to 2 years), seasonal isotopic variability will be incorporated into the tissue. Where the “source” used in the model was based on white sucker tissue (e.g. the upper estuary), the model accounts for this variability. However, the model does not account well for seasonal variability in Lake Superior because we used stable isotope values from prey sampled during the summer months. In Lake Michigan, the seasonal isotopic change in seston at the base of the food web is approximately 6 % for both δ^13^C and δ^15^N; the seston is enriched in the heavy isotopes during the summer months (McCusker et al. 1999). In contrast, the spatial distribution of ^13^C and ^15^N along the river–lake continuum in the St. Louis River AOC was found to vary seasonally, as a function of river discharge, but the magnitude of difference between lake and river varied little (Hoffman et al. 2012). Second, the model assumes fish generally assume the same trophic level; however analysis of white sucker diets has demonstrated selective feeding (Saint Jacques et al. 2000). Thus, it is plausible that some of the δ^15^N variation is a result in variability in trophic level. If so, the effect on the model would be greatest on lower estuary contributions. White sucker feeding in the lower estuary at a relatively low trophic levels would be assigned a mix of lake and upper estuary to account for the lower δ^15^N in the fish. Trophic fractionation can also vary with respect to diet, food quality, and metabolic state (Gannes et al. 1997; Vander Zanden and Rasmussen 2001; Caut et al. 2009). A detailed study of these factors was outside the scope of this study, however, the lake and estuary were well-separated by δ^13^C, suggesting estimates are less sensitive to trophic level and trophic fractionation effects.

In conclusion, the overall incidence of both skin and liver tumors was low (4.5 %), although raised skin lesions, potentially preneoplastic, were high (31 %). Liver tumors (all of bile duct origin) and the incidence of putative preneoplastic foci of cellular alterations were also low (4.5 %). Further research is required to determine both chemicals and biological risk factors for skin and liver tumors of white sucker. The logistic regression model indicated that white sucker older than 9 years had more than twice the skin tumor incidence of the general population. In addition, when controlling for age, white sucker that derived more than half their diet from the upper estuary were 1.7 times more likely to have skin lesions than those that derived most of their diet from the lower estuary and Lake Superior. Hence, stable isotopes as a screening tool may be useful in determining habitat-related effects and as such, assist in targeting remediation and restoration efforts.
